# Supplementary material for: Assessment of Nanopollution from Commercial Products in Water Environments
Source: Nanomaterials (Basel). 2021 Sep 28;11(10):2537. doi: 10.3390/nano11102537 (PMC8539925; doi:10.3390/nano11102537)
Supplement: Supplementary file 1 [file nanomaterials-11-02537-s001.zip › nanomaterials-1373172-supplementary.pdf]

nanomaterials

MDPI

Supplementary

Assessment of Nanopollution in Water Environments from Commercial Products

Raisibe Florence Lehutso [1,2] and Melusi Thwala [1,3,4,*]

[1] Water Centre, Council for Scientific and Industrial Research, Pretoria, 0001, South Africa; flehutso@csir.co.za
[2] Department of Chemical Sciences, University of Johannesburg, Johannesburg, 2028, South Africa
[3] Department of Environmental Health, Nelson Mandela University, Gqeberha, 6019, South Africa
[4] Centre for Environmental Management, University of the Free State, Bloemfontein, 9031, South Africa
* Correspondence: mthwala@csir.co.za

Properties of the release media of SUN1–3

For artificial freshwater, commercial Valpré spring water, sourced from the Fricona Valley in the highlands of northern KwaZulu-Natal, South Africa, was purchased from a local store (Clicks, South Africa). The mineral composition of Valpré Spring water was [Calcium = 10 mg/L, Magnesium = 10 mg/L, Sodium = 3 mg/L, Potassium = 1 mg/L, Chloride = 2mg/L, Sulphate = 4 mg/L, alkalinity (as $CaCO_3$) = 65 mg/L, Nitrate = 1 mg/L, Fluoride = 0.1 mg/L, and total dissolved solid = 83 mg/L. Artificial seawater was prepared from commercial Red sea salt (Jungle Aquatics, South Africa); the solution was prepared for parameters of 35 ppt [1]. Artificial swimming pool water was prepared in Milli-Q water (MQ) and was made up of $CaSO_4{\cdot}2H_2O$, $NaHCO_3$ (both purchased from Merck, South Africa) and NaClO (3.5%) (Clicks, South Africa) were prepared following a previously reported procedure [2,3].

Table S1. Average physicochemical properties of release media before and after ENMs release.

	pH	Conductivity (µs/cm)	Turbidity (NTU)
**Before release**			
Milli-Q water	8.61 ± 0.04	1.71 ± 0.03	0.24 ± 0.01
Freshwater	7.70 ± 0.09	148.5 ± 0.08	0.19 ±0.02
Seawater	5.08 ± 0.01	41400 ± 100	1.90 ± 0.01
Swimming pool water	5.78 ± 0.08	5139 ± 89	237 ± 7
**After release**			
Milli-Q water–SUN1	8.56 ± 0.4	112.3 ± 5	5.67 ± 0.4
Freshwater –SUN1	7.53 ±0.09	249 ± 4	5.98 ± 0.9
Seawater –SUN1	6.78 ±.0.1	64.9 ± 1	5.61 ± 0.3
Swimming pool water –SUN1	6.71 ± 0.09	7.02 ±2	3.52 ± 0.1
Milli-Q water –SUN2	8.59 ± 0.17	237 ± 7	20.4 ± 2
Freshwater –SUN2	7.58 ± 0.13	277.0 ± 5	61.9 ± 2
Seawater –SUN2	6.69 ± 0.09	64 ± 3	89.8 ± 1
Swimming pool water –SUN2	7.24 ± 0.1	6.88 ± 1	58.2 ± 2
Milli-Q water –SUN3	8.26 ± 0.05	185.2 ± 3	66.8 ± 3
Freshwater –SUN3	7.73 ± 0.1	288 ± 4	74.9 ± 5
Seawater –SUN3	6.84 ± 0.07	62.4 ± 1	48.1 ± 1
Swimming pool water –SUN3	7.40 ±0.12	7.00 ± 1	71.1 ± 2
Milli-Q water –CA1	7.23 ± 0.15	9.95 ± 2	80.8 ± 1
Milli-Q water –SAN1	8.43 ± 0.09	3.83 ± 0.1	0.42 ± 0.02
Milli-Q water –SK1	7.71 ± 0.12	81.0 ± 1	40.2 ± 2

Physicochemical properties in SUN1–3 and CA1 release media under light and dark conditions were not significant.

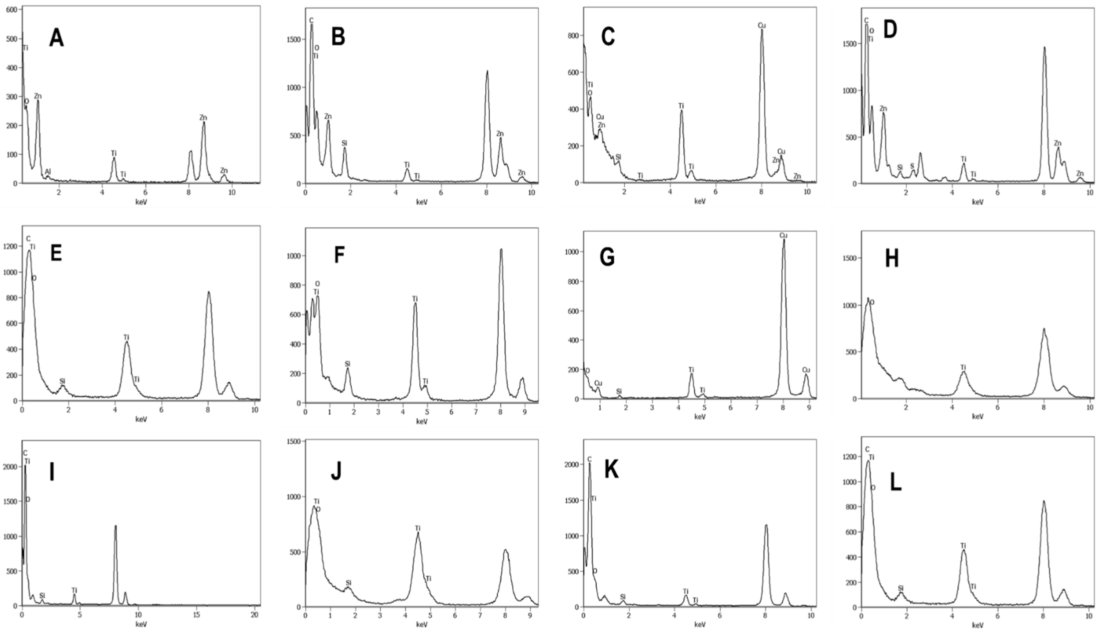

Figure S1. The EDX spectras' of product released–ENMs obtained under light conditions for SUN1 detected in milli-Q water (A), freshwater (B), swimming pool water (C), seawater (D); SUN2 detected in milli-Q water (E), freshwater (F), swimming pool water (G), seawater (H) and SUN3 detected in milli-Q water (I), freshwater (J), swimming pool water (K), seawater (L).

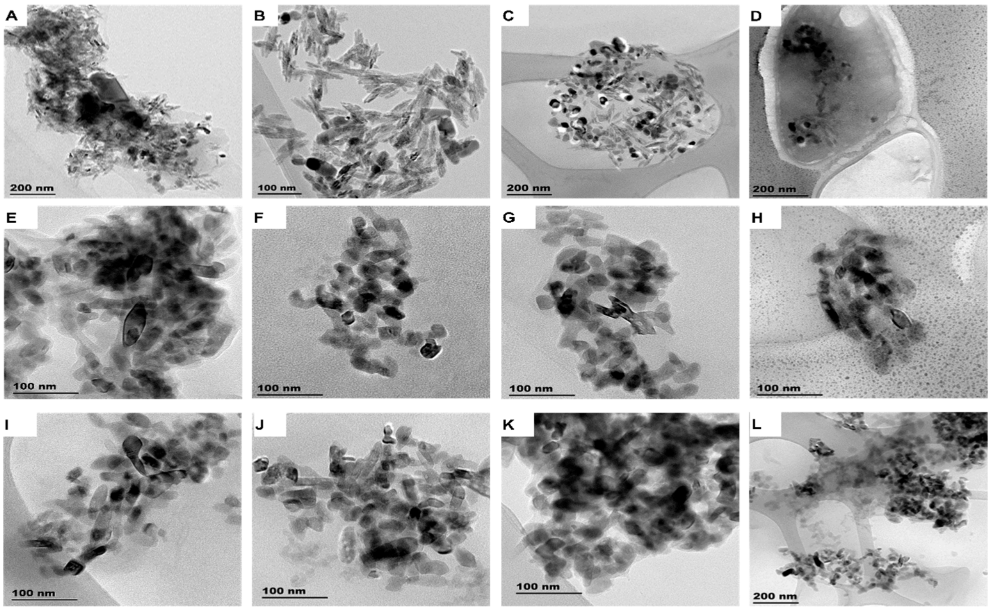

Figure S2. TEM images of product released–ENMs obtained under dark conditions for SUN1 detected in milli-Q water (A), freshwater (B), swimming pool water (C), seawater (D); SUN2 detected in milli-Q water (E), freshwater (F), swimming pool water (G), seawater (H) and SUN3 detected in milli-Q water (I), freshwater (J), swimming pool water (K), seawater (L).

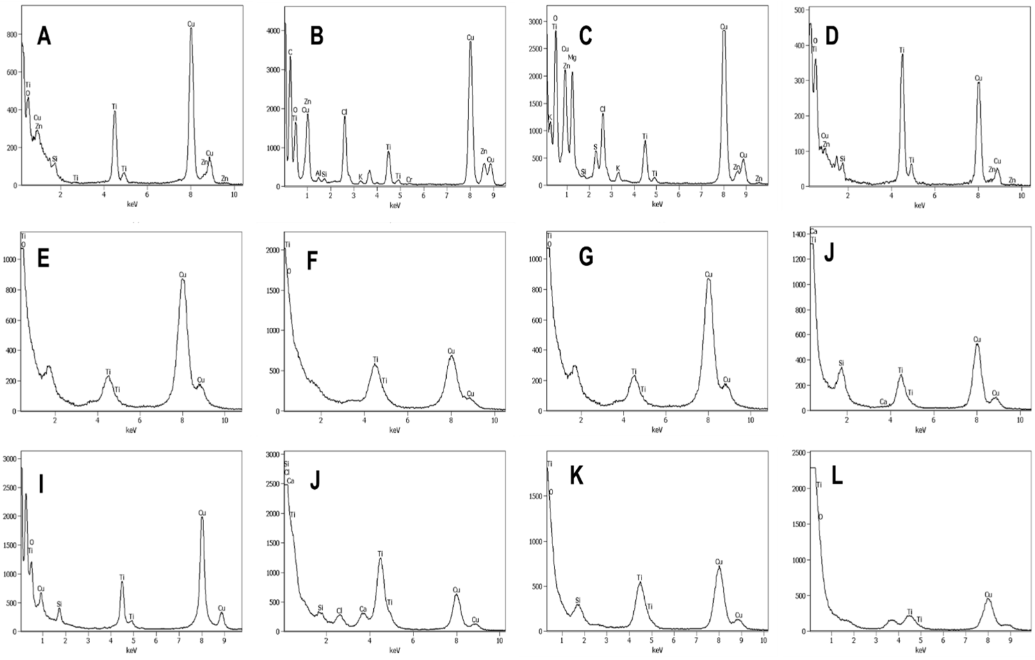

Figure S3. Corresponding EDX images of product released–ENMs obtained under dark conditions for SUN1 detected in milli-Q water (A), freshwater (B), swimming pool water (C), seawater (D); SUN2 detected in milli-Q water (E), freshwater (F), swimming pool water (G), seawater (H) and SUN3 detected in milli-Q water (I), freshwater (J), swimming pool water (K), seawater (L).

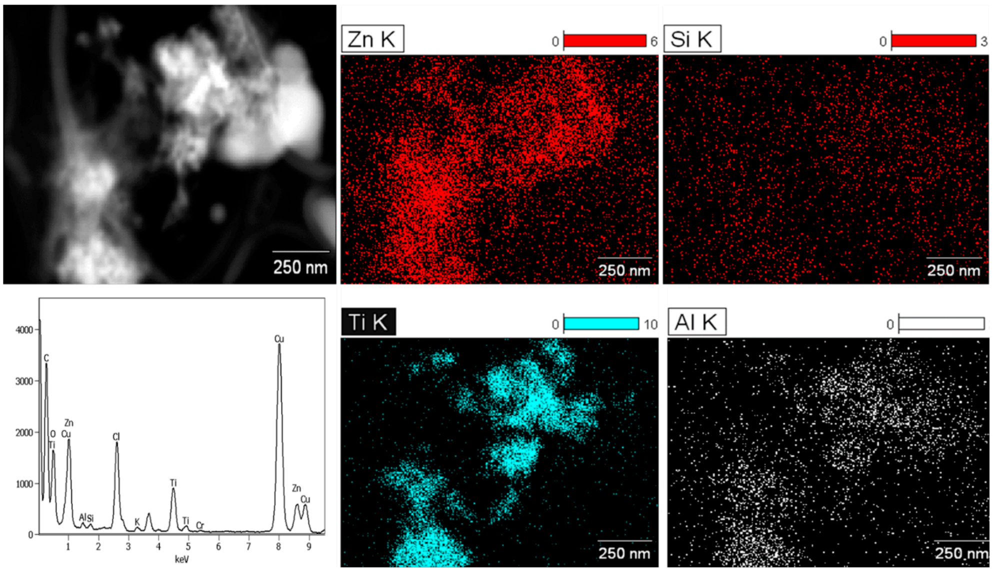

Figure S4. EDX elemental mapping showing adsorption and desorption of ENMs coating agents (Si and Al) on SUN1 released–ENMs.

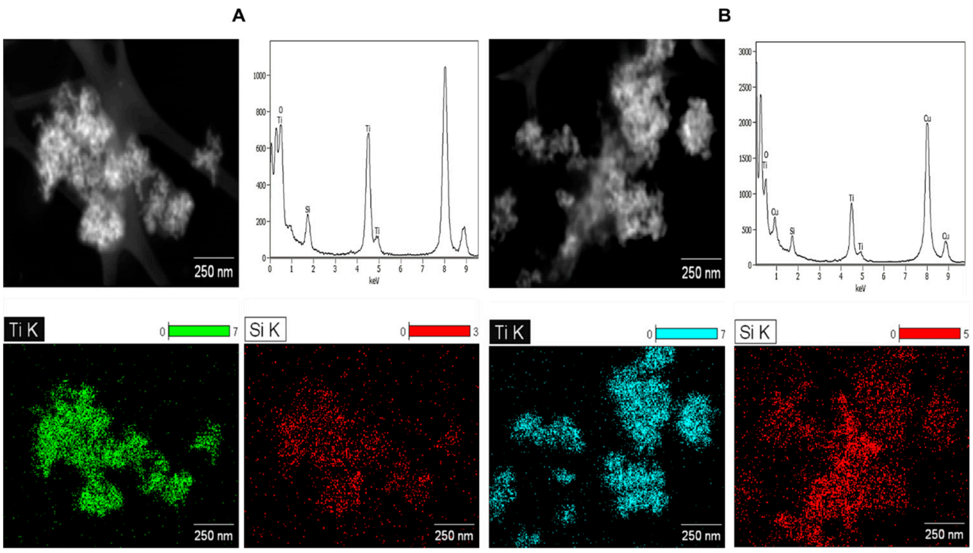

Figure S5. EDX elemental mapping showing adsorption and desorption of ENMs coating agents (Si) on SUN2 (A) and SUN3 (B) released–ENMs.

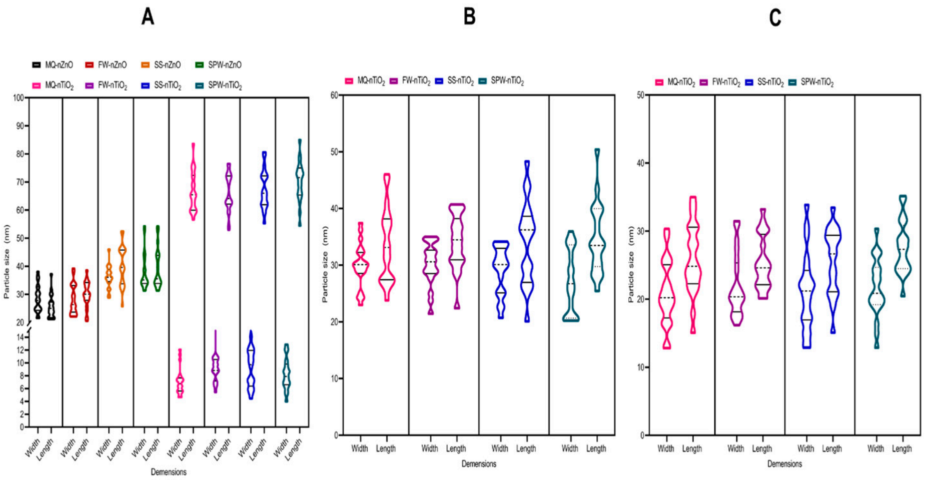

Figure S6. A. Violin plot showing particle distribution of SUN1(A), SUN2 (B), and SUN3 (C) released–ENMs obtained under light conditions. Upper and lower quartiles are highlighted by a solid line, while the dotted line indicates the median. The denser the violin shape the higher the number of the particle size in that region.

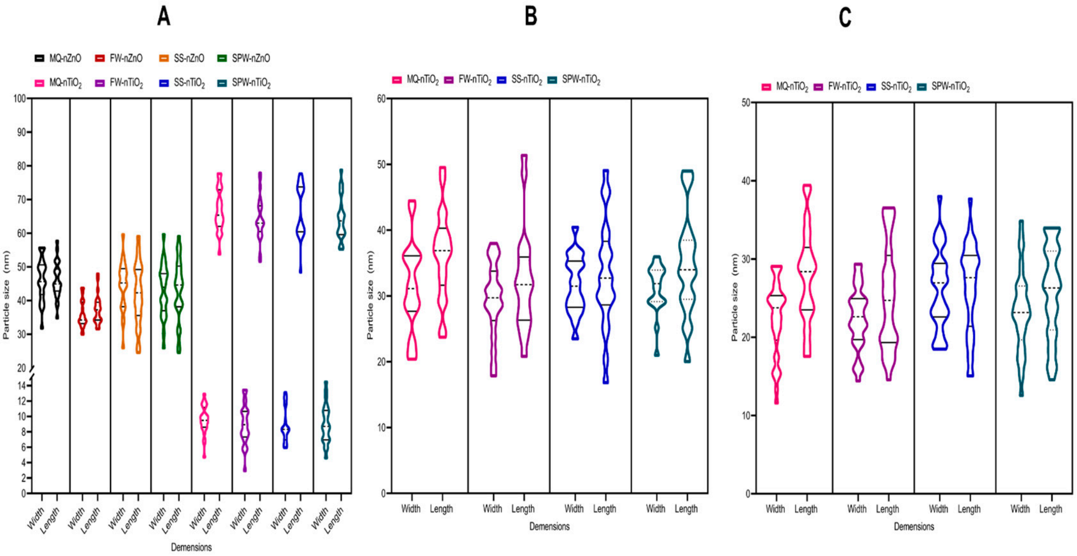

Figure S6. B. Violin plot showing particle distribution of SUN1(A), SUN2 (B), and SUN3 (C) released–ENMs obtained under dark conditions. Upper and lower quartiles are highlighted by a solid line, while the dotted line indicates the median. The denser the violin shape, the higher the number of particle size in that region.

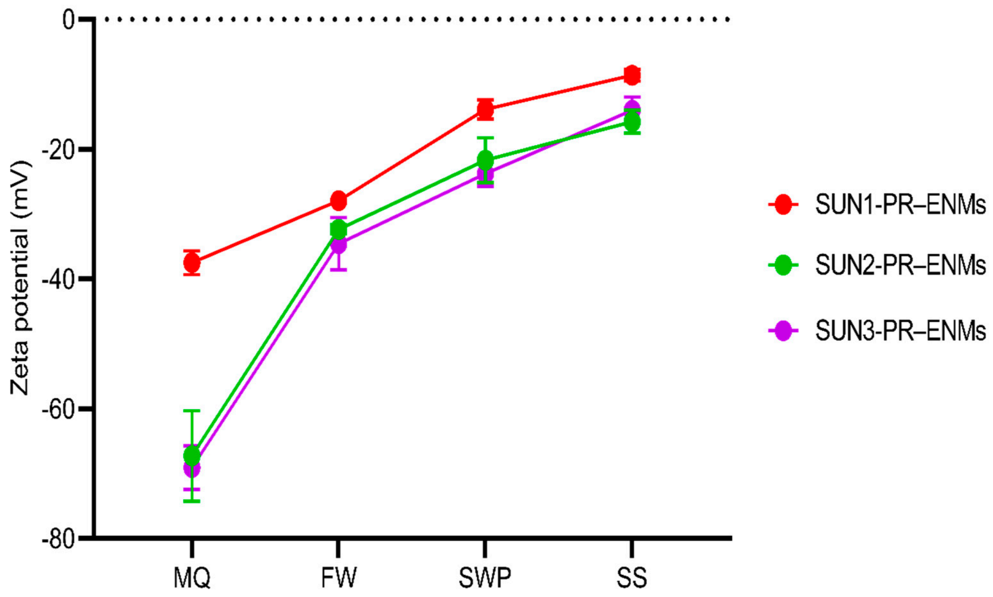

Figure S7. Zeta potential of SUN1–3 released–ENMs obtained under dark conditions in different release media of milli-Q water (MQ), freshwater (FW), swimming pool water (SPW), and seawater (SS).

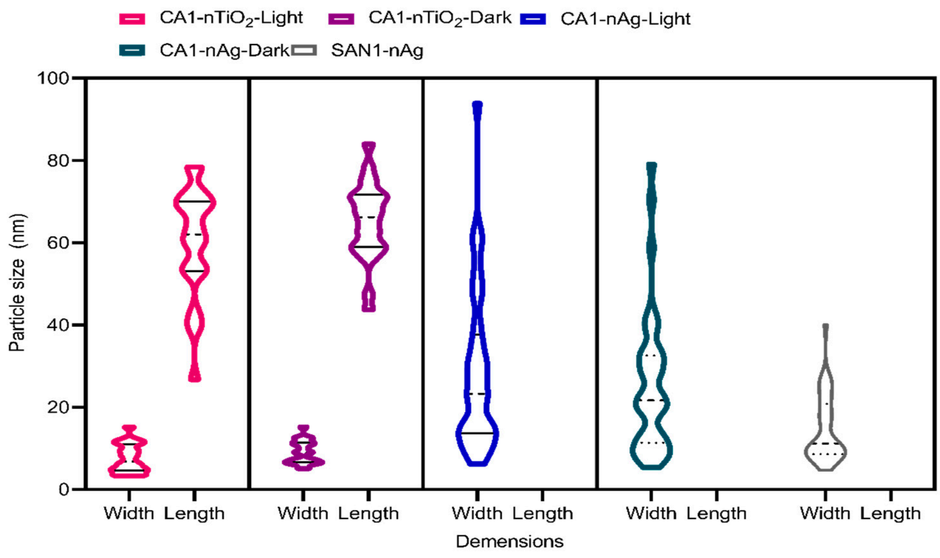

Figure S8. Violin plot showing particle distribution of CA1 released–ENMs (obtained under light and dark conditions) and SAN1 released–ENMs. Upper and lower quartiles are highlighted by a solid line, while the dotted line indicates the median. The denser the violin shape the higher the number of the particle size in that region.

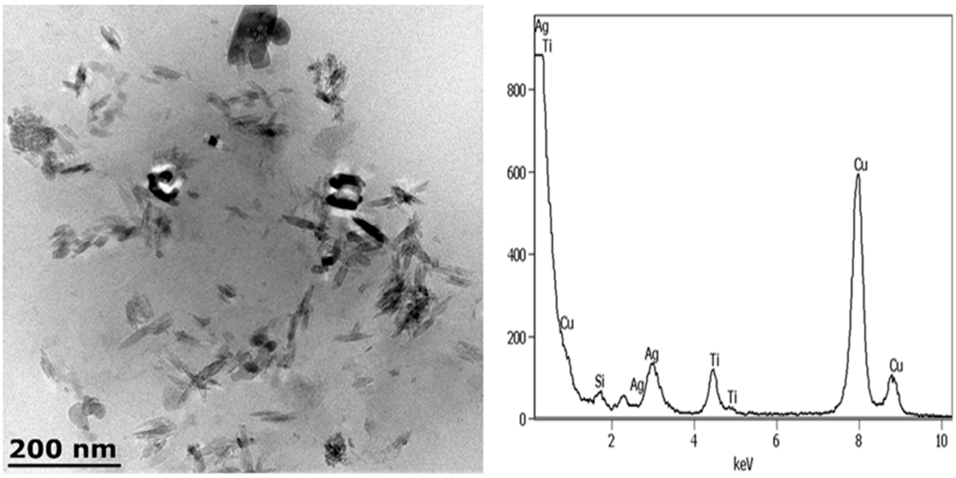

Figure S9. TEM-EDX image showing of CA1 product released–nAg and product released–$nTiO_2$ obtained under dark conditions.

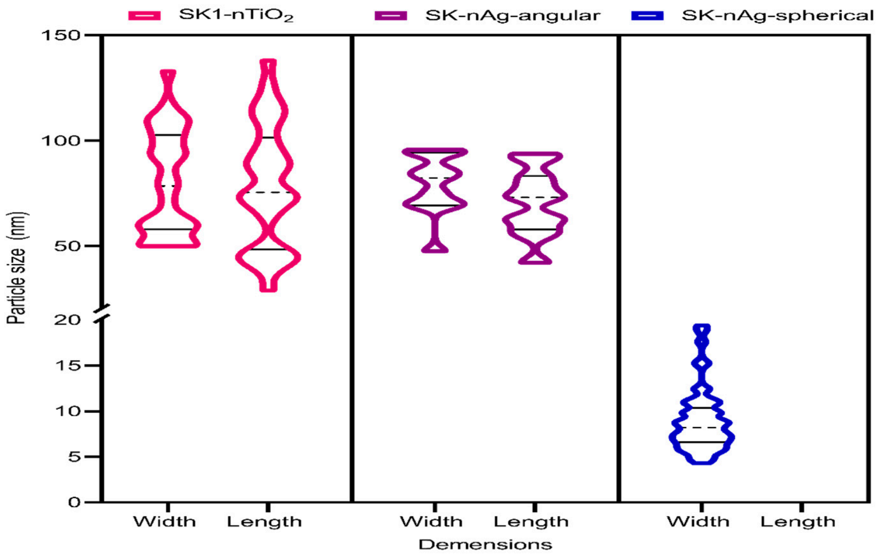

Figure S10. Violin plot showing particle distribution SK1 released–ENMs. Upper and lower quartiles are highlighted by a solid line, while the dotted line indicates the median. The denser the violin shape, the higher the number of particle size in that region**.**

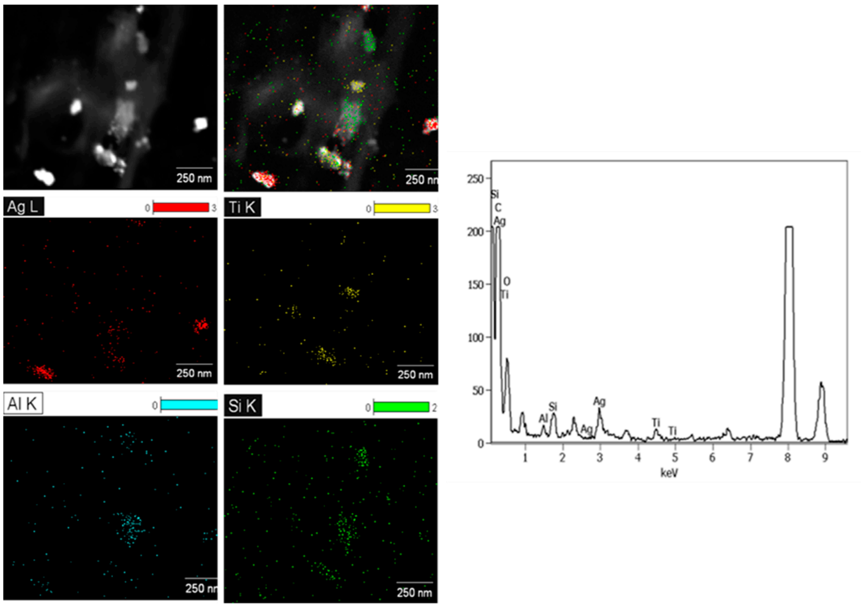

Figure S11. Elemental mapping of binary SK1 released–ENMs identified as product released–$nTiO_2$ (yellow) and product released–nAg (red). The images further show evidence of SK1 released–$nTiO_2$ particles partially still coated with Si and Al.

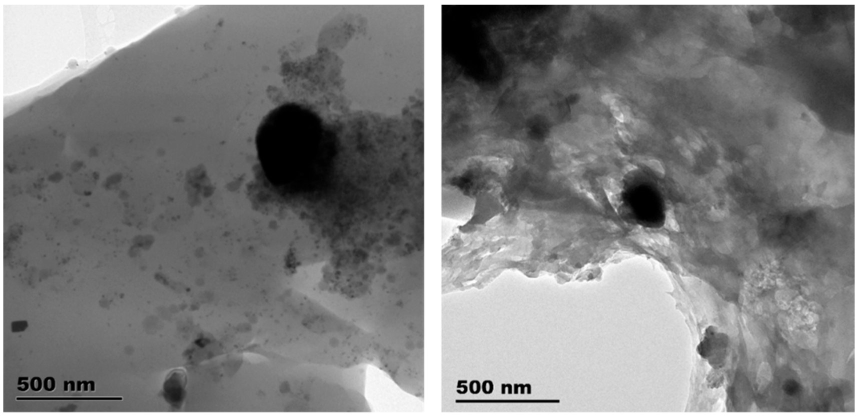

Figure S12. TEM images showing the thick layer introduced by washing SK1 with sodium dodecyl sulfate release media**.**

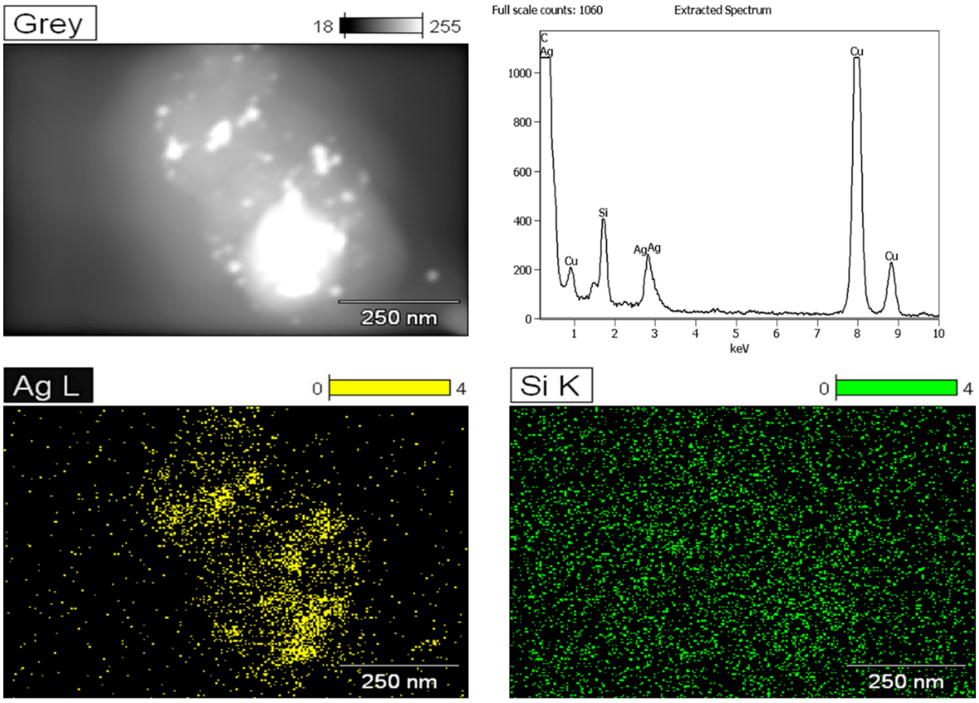

Figure S13. EDX elemental mapping illustrating Si desorbed from CA1 released–ENMs.

References

1. Red Sea Salt -Ideal for mature reef and low nutrient systems Available online: https://www.redseafish.com/red-sea-salts/red-sea-salt/ (accessed on Mar 23, 2020).

2. Anipsitakis, G.P.; Tufano, T.P.; Dionysiou, D.D. Chemical and microbial decontamination of pool water using activated potassium peroxymonosulfate. *Water Res.* **2008**, *42*, 2899–2910, doi:10.1016/j.watres.2008.03.002.

3. Virkutyte, J.; Al-Abed, S.R. Statistical evaluation of potential damage to the Al(OH)3 layer on nTiO2 particles in the presence of swimming pool and seawater. *J. Nanoparticle Res.* **2012**, *14*, 787, doi:10.1007/s11051-012-0787-7.
